# Impact VR: A Socioemotional Intervention for Reducing CU Traits, Conduct Problems, and Aggression in Youth with Conduct Disorder

**DOI:** 10.1007/s10802-025-01373-3

**Published:** 2025-10-07

**Authors:** Nicholas D. Thomson, Robert A. Perera, Salpi S. Kevorkian, Laura Hazlett, Scott Vrana

**Affiliations:** 1 Arche XR LLC, 2400 Old Brick Road, Glen Allen, VA 23060 USA; 2 Department of Surgery, Virginia Commonwealth University, Richmond, VA USA; 3 Department of Psychology, Virginia Commonwealth University, Richmond, VA USA; 4 Department of Biostatistics, Virginia Commonwealth University, Richmond, VA USA; 5 Department of Criminology and Criminal Justice, Florida International University, Miami, FL USA

**Keywords:** Callous-unemotional traits, Conduct disorder, Reactive aggression, Proactive aggression, Intervention, Prevention, Virtual reality

## Abstract

Conduct Disorder (CD) and callous-unemotional (CU) traits are associated with persistent antisocial behavior, emotional processing deficits, and poor treatment response. CU traits designate a subgroup of youth with CD who are at greater risk of violence and long-term mental health challenges. It is well-established that CU traits, CD, and aggression are tied to deficits in emotion recognition, social information processing, and interpersonal functioning, yet few interventions directly target these mechanisms. This randomized controlled trial tested the effects of Impact VR, a brief virtual reality program designed to improve emotion recognition and build social-emotional skills in youth with CD. One hundred and ten youth diagnosed with CD were randomly assigned to either Impact VR or a treatment control group. Youth and caregivers completed assessments at baseline, post-intervention, and 3-month follow-up. Youth in the Impact VR group had lower levels of self-reported and caregiver-reported CU traits at follow-up. Caregiver ratings also indicated that youth who received Impact VR had significantly lower conduct problems at both follow-up time points. Youth reported immediate reductions in reactive aggression, which was sustained until the 3-month follow-up. However, the intervention groups did not significantly differ on proactive aggression. These findings suggest that CU traits may be modifiable through brief, engaging interventions like Impact VR.

Conduct disorder (CD) is one of the most common and impairing psychiatric conditions in children and adolescents (Fairchild et al., 2019; Kessler et al., 2012; Lindhiem et al., 2015). With a global prevalence of 3.2%, CD is associated with more than 5.75 million years of healthy life lost worldwide (Erskine et al., 2014). Youth with CD are more likely to need services from mental health, school, and legal systems. Some studies estimate that CD costs over $70,000 per child across a seven-year period, largely due to expenses from mental health treatment, education services, and juvenile justice involvement (Knapp et al., 2011). Other long-term studies confirm that CD places a heavy burden on public resources well into adulthood (Rivenbark et al., 2018). Children with CD are more likely to experience legal problems, substance use, social difficulties, and chronic mental and physical health issues across their lifespan (Jones, 2013; Odgers et al., 2008).

Due to the long-term effects of CD, there is a need to identify risk factors and underlying mechanisms that can inform treatment. To improve care planning and account for the variability in CD presentations, the DSM-5 introduced a specifier called “Limited Prosocial Emotions,” which reflects the presence of callous-unemotional (CU) traits (American Psychiatric Association, 2022). Between 12 and 46% of youth with CD meet the criteria for significant CU traits (Edwards et al., 2018; Ermer et al., 2012; Fanti, 2013, 2018; Pardini et al., 2012; Thomson et al., 2024). These youth exhibit the same core symptoms of CD but also show a lack of empathy, shallow or insincere emotional responses, and little concern for others or their own performance (Blair et al., 2014; Kimonis et al., 2014). Youth with CU traits are more likely to engage in more severe violence and chronic antisocial behavior and to continue experiencing serious adjustment problems into adulthood (Colins & Andershed, 2015; Frick et al., 2014b; Rowe et al., 2010). The presence of CU traits signals a group of youth with CD who are more resistant to treatment and more likely to remain impaired over time.

Although CU traits can help guide treatment selection and goals (Frick & Nigg, 2012), only a few available treatments specifically address CU traits. Most treatments focus on behavior management rather than the emotional or interpersonal deficits contributing to CU traits (Thomson et al., 2017). The most evidence-based treatments for conduct problems include intensive family-focused interventions such as multisystemic therapy (MST) and functional family therapy (FFT). These treatments are effective at reducing criminal activity and antisocial behavior in youth with conduct problems and CU traits (Borduin et al., 1995; White et al., 2013), but they have not consistently reduced CU traits or changed how these youth process emotional information. Further, due to the intensive approach to treatment, they are expensive to implement, costing between $5,000 and $10,000 per child annually (Sheidow et al., 2004). Although they may produce long-term savings in reduced service use, the initial investment is often out of reach for schools and mental health systems. There remains a gap in effective, accessible treatments that can directly improve CU traits and the underlying impairments that contribute to them, especially treatments for adolescents.

One promising approach is to target emotion recognition difficulties, which are commonly seen in youth with CD and CU traits. These youth often struggle to recognize facial expressions and vocal cues, especially those related to fear and sadness (Dawel et al., 2012; White et al., 2016). These deficits limit their ability to interpret social situations, build empathy, and respond appropriately to others' distress. Neuroimaging studies show that youth with CU traits have reduced activity in the amygdala and weaker connectivity with brain regions involved in emotional learning and regulation (Marsh et al., 2008). Emotion recognition deficits are not unique to CD. They are also observed in youth with other psychiatric conditions, including oppositional defiant disorder and ADHD (Kleine Deters et al., 2020; Staff et al., 2022). This suggests that emotion recognition difficulties may be a transdiagnostic issue that contributes to social impairment across childhood disorders. Because CD is often comorbid with other disorders, targeting emotion processing may improve functioning across domains and make treatment more impactful.

There is growing interest in improving emotion recognition through direct intervention. There is evidence that emotion recognition can improve with training, even in youth with severe behavior problems and CU traits. In one study, youth were taught to pay closer attention to the eye region of faces, which led to better recognition of fear and sadness and improved social behavior (Dadds et al., 2012). Improvements in emotion recognition were maintained over time, even among youth with high levels of CU traits (Muñoz Centifanti et al., 2021). Other studies using computerized emotion training programs found reductions in criminal behavior and improvements in empathy and conduct (Hubble et al., 2015; Wells et al., 2021). While these findings are promising, there remains little evidence that these strategies work for reducing CU traits and conduct problems among youth with CD.

One of the biggest challenges in treating youth with CD and CU traits is engagement (Thomson et al., 2025a, b). Estimates of treatment dropout range between 40 and 60%, with even higher rates among youth with externalizing disorders (Kazdin et al., 1997; Hawes & Dadds, 2005; Thomson et al., 2025a, b). Even the most well-established interventions, such as MST and FFT, require intensive and multi-system involvement and still face high dropout rates (Sheidow et al., 2004). Research has also shown that youth with CU traits may be particularly resistant or difficult to engage in interventions, as they often exhibit low motivation for change and limited concern for social consequences (Frick et al., 2014a; Washburn et al., 2007). In a recent study involving youth with CD, over 90% reported that they disliked attending therapy, describing it as boring, irrelevant, or judgmental (Thomson et al., 2025a, b). Many said they had avoided or skipped sessions, and 68% had run away or become unreachable to avoid appointments. These findings suggest that poor engagement is not limited to specific treatment types but reflects a broader mismatch between traditional therapy formats and the preferences, needs, and developmental profiles of this group.

In addition to low engagement, youth with CD face several barriers to treatment that limit access and effectiveness. Many youth report feeling stigmatized by their involvement in therapy, particularly when services are visible to peers or occur in settings like schools or juvenile justice programs. In the same study, 90% of youth felt embarrassed or ashamed when receiving case management services (Thomson et al., 2025a, b). Eighty-four percent believed therapists could not relate to their daily challenges, and 80% said they felt negatively judged during treatment. Families face challenges as well. Over 80% of parents reported dissatisfaction with their child's treatment, with the most common complaints including long wait times (ranging from 2 to 16 months), lack of school-based resources, and minimal improvements in their child’s behavior. Every parent in the sample reported that their child had at some point avoided or run away from therapy. These barriers, which included stigma, accessibility, system overload, and lack of youth engagement, compound to create a treatment environment that is poorly aligned with the needs of this population. As a result, traditional interventions often fall short because they fail to meet the attention demands, motivational characteristics, and real-world constraints that youth with CD and CU traits experience (Thomson et al., 2025a, b). To improve youth outcomes, treatments must be designed to address underlying mechanisms, as well as to increase youth engagement and reduce barriers to accessing care.

Impact VR is a brief, immersive intervention designed to improve emotion recognition skills in youth with CD and CU traits through engaging psychoeducation, gamified emotion training, and virtual social simulations. The program places youth as problem solvers in emotionally salient interpersonal conflict scenarios where they identify emotional expressions in others and make decisions that support prosocial responses. Each module includes targeted feedback and repetition to reinforce learning, while the immersive format is designed to enhance engagement. Grounded in research linking CU traits to emotion recognition deficits, Impact VR was co-developed with a youth advisory group with similar experiences (youth with CD) and refined through extensive input from mental health clinicians, educators, and caregivers of youth with CD. This participatory design process led to a program that was rated as highly acceptable, feasible, and appropriate across multiple stakeholder groups (Thomson et al., 2025a, b). Youth reported positive experiences, with 100% stating that Impact VR was a good use of their time and preferred it over traditional mental health services (e.g., therapy, counseling, or case management), and felt that the program was culturally sensitive and avoided stereotypes, and all participants (100%) said they would recommend the program to a friend (Thomson et al., 2025a, b). Most youth (95%) reported learning new skills during Impact VR, and 100% said they felt more confident recognizing emotions in others. Youth also reported that the skills learned through the program would improve their relationships with friends (100%), parents (90%), and teachers (90%). Importantly, higher CU traits were associated with higher acceptability ratings, suggesting that the VR format may be especially engaging for youth who are typically resistant to traditional forms of care (Thomson et al., 2025a, b). These preliminary findings suggest that Impact VR is not only developmentally appropriate but may also fill a critical gap for youth who are underserved by existing interventions. Further, among youth with CD, Thomson et al. (Thomson et al., 2025b) found Impact VR improved emotion recognition accuracy and interpersonal relationships among peers and parents compared to the treatment control group. Although Impact VR has demonstrated encouraging results for improving social skills, Impact VR has not yet been evaluated using a randomized controlled trial (RCT) to test its effectiveness for reducing CU traits and conduct problems.

The present study tested the effectiveness of Impact VR in an RCT with a sample of youth diagnosed with CD. The aim was to evaluate whether Impact VR led to reductions in CU traits, conduct problems (CP), and both reactive and proactive aggression. Outcomes were assessed at two time points: immediately post-intervention (4 weeks after baseline) and at a 3-month follow-up. Youth were randomly assigned to either Impact VR or a comparative treatment control condition. We hypothesized that, compared to the control group, youth receiving Impact VR would demonstrate greater reductions in CU traits (reported by both youth and caregivers), CP (reported by caregivers), and aggression subtypes (reported by youth).

## Methods

### Participants

Participants were recruited from a large, urban healthcare network in Virginia. All participants had a current CD diagnosis made by a licensed mental health professional. A total of 110 participants aged 10–17 (*M*_*age*_ = 13.79, *SD* = 2.33) years old were included in this study. Participants were 59% male and 41% female, and self-identified as African American (55%), White (38%), or other (7%). Participant demographics and mean scores on baseline variables are displayed in Table 1. Examination of participant characteristics revealed no meaningful differences between groups on CU, CP, or aggression subtypes.

**Table 1:** Descriptives for full sample and intervention groups

	Full Sample (*N* = 110)	Impact VR (*n* = 55)	Control Group (*n* = 55)
Mean age (*SD*)	13.79 (2.33)	13.91 (2.27)	13.69 (2.43)
Sex			
Male	64 (58%)	33 (60%)	31 (56%)
Female	46 (42%)	22 (40%)	24 (44%)
Ethnicity*			
Black	64 (58%)	33 (60%)	31 (56%)
White	36 (33%)	18 (32%)	19 (34%)
Other	10 (9%)	4 (8%)	5 (10%)
Comorbid Diagnoses			
ADHD	36 (33%)	20 (36%)	17 (30%)
Autism	11 (10%)	7 (12%)	4 (8%)
Anxiety Disorder	14 (13%)	8 (14%)	7 (12%)
Post-Traumatic Stress Disorder	11 (10%)	4 (8%)	7 (12%)
Major Depressive Disorder	18 (16%)	10 (18%)	8 (14%)
Mean Baseline Scores (*SD*)			
CU traits (SR)	25.60 (9.30)	25.96 (9.28)	25.02 (9.36)
CU traits (CR)	27.89 (12.37)	28.05 (11.73)	27.21 (12.61)
Conduct Problems (CR)	57.14 (15.35)	58.89 (15.71)	54.85 (14.52)
Reactive Aggression (SR)	10.44 (4.61)	11.14 (4.44)	9.70 (4.74)
Proactive Aggression (SR)	2.38 (3.07)	2.27 (2.83)	2.36 (3.18)

^*^Other = Asian, Native American, Pacific Islander

*SR* Self-Report; *CR* Caregiver-Report

### Procedure

Participant eligibility included being 10–17 years old, English-speaking, and diagnosed with CD. CD diagnosis was made by a licensed clinician. Participants with a current CD diagnosis were recruited from a large, urban healthcare network in Virginia. Caregiver–youth dyads were contacted by email and phone, and were provided information about the study. Interested participants were invited into the lab. Before study participation, youth provided assent, and caregivers provided consent. Assent and consent were conducted in different and private rooms. Caregiver and youth were informed about the randomization procedure prior to consent/assent. Next, youth participants completed surveys about themselves, which took about 45 min. Caregivers completed assessments about the child in a separate interview room. Once the baseline assessment was completed, participants were randomly assigned to either the Impact VR or treatment control groups. To achieve equal sample size in each group, block randomization was used with random block sizes of 4, 6, and 8. Block randomization is recommended for smaller sample sizes (Efird, 2011). The participant and research coordinator conducting the baseline assessment were blinded to the randomization order until after the baseline assessment. This approach maintained assessor blinding during baseline and reduced the potential for allocation bias. Youth in the treatment control group completed a training program on identifying emotional expressions in others, similar to prior research (Dadds et al., 2012; Muñoz Centifanti et al., 2021). Youth in the Impact VR group completed the first of four weekly sessions. Sessions 2, 3, and 4 of Impact VR were completed in the home of the youth. Thus, participants in the intervention condition completed four weekly 25-min sessions, while those in the active control condition completed a single 25-min session. Follow-up assessments were completed online or in person. Follow-up assessments were conducted at two time points: 1-month and 3-months post-randomization. The first follow-up occurred approximately 4 weeks after baseline, immediately following the final session of Impact VR, and is referred to as post-intervention timepoint. Youth and caregiver participants received $75 each for completing each assessment. No changes in the protocol or methods occurred during the clinical trial period. This randomized controlled trial was preregistered at ClinicalTrials.gov (NCT06301516) and approved by the Virginia Commonwealth Institutional Review Board.

### Interventions

#### Treatment Control

Rather than assigning participants to a passive control condition, the study utilized an active comparator designed to replicate prior one-time brief emotion recognition training procedures previously shown to improve emotion recognition among youth high in CU traits (see Dadds et al., 2012; Muñoz Centifanti et al., 2021). Youth in the control condition participated in a single, 25-min emotion recognition training session. This session was structured as a psychoeducational slideshow facilitated by a study coordinator. The training was divided into two instructional segments. The first segment focused on *where* to look when identifying emotions, emphasizing that emotional cues are primarily found in the eyes and mouth. Participants were presented with a series of illustrated slides for four core emotions: happiness, sadness, anger, and fear. Each emotion was paired with visual prompts directing attention to the facial regions most relevant for emotional expression. The second segment instructed participants *how* to recognize each emotion by describing specific muscular and facial movement patterns associated with each expression. For example, fear was described as involving raised eyebrows, wide-open eyes, and a dropped jaw, while sadness involved drooping eyes, a downturned mouth, and sloped eyebrows. The session concluded with a summary slide encouraging participants to always observe the eyes and mouth to determine what another person may be feeling. To promote engagement and retention, the coordinator asked each participant to describe where they would look on a person’s face to understand how they were feeling. The coordinator gave corrective feedback as needed. This format was designed to match prior protocols.

#### Impact VR

**Fig. 1:**
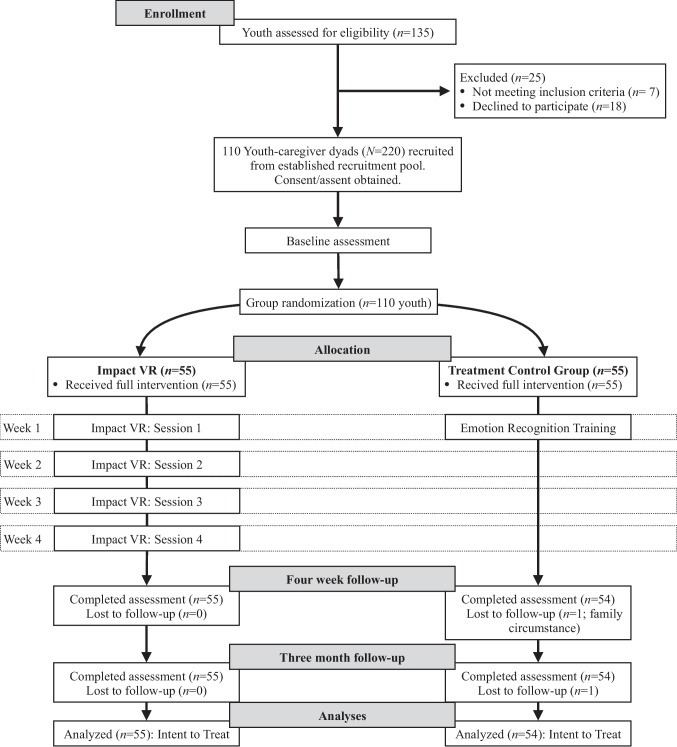
CONSORT diagram of study

Impact VR (Arche XR, 2024) is an immersive virtual reality intervention aimed at enhancing social-emotional learning by targeting emotion recognition, emotion regulation, and prosocial behavior. The program utilizes 360-degree technology to place youth in emotionally meaningful, interactive scenarios to simulate real-world social situations. Grounded in cognitive-behavioral and dialectical behavior therapy frameworks, the intervention integrates psychoeducation with gamified, experiential learning strategies to support skill acquisition and emotional development. Participants are guided through a series of emotion recognition tasks that involve interpreting facial expressions under varying conditions, including when cues are partially obscured (e.g., by sunglasses or masks). In parallel, youth are taught to identify emotional triggers and develop strategies to manage challenging affective states. The VR platform provides real-time feedback and dynamically adapts task difficulty to sustain engagement, minimize frustration, and prevent performance plateaus. Social problem-solving activities are embedded within a narrative structure, allowing youth to practice interpreting others’ emotional states, identifying underlying causes of emotional reactions, and choosing prosocial responses to interpersonal conflict. Consistent with research by Mancuso et al. (2023), the emotionally immersive nature of VR promotes ecological validity and enhances memory consolidation, increasing the likelihood that skills learned within the program will generalize to real-life contexts. Impact VR is fully self-guided and can be used independently or with support from a clinician. Its flexible design allows for implementation across various settings, including clinics and community-based environments, and is particularly well-suited for populations with limited access to traditional therapeutic services (Fig. 1).

**Fig. 2:**
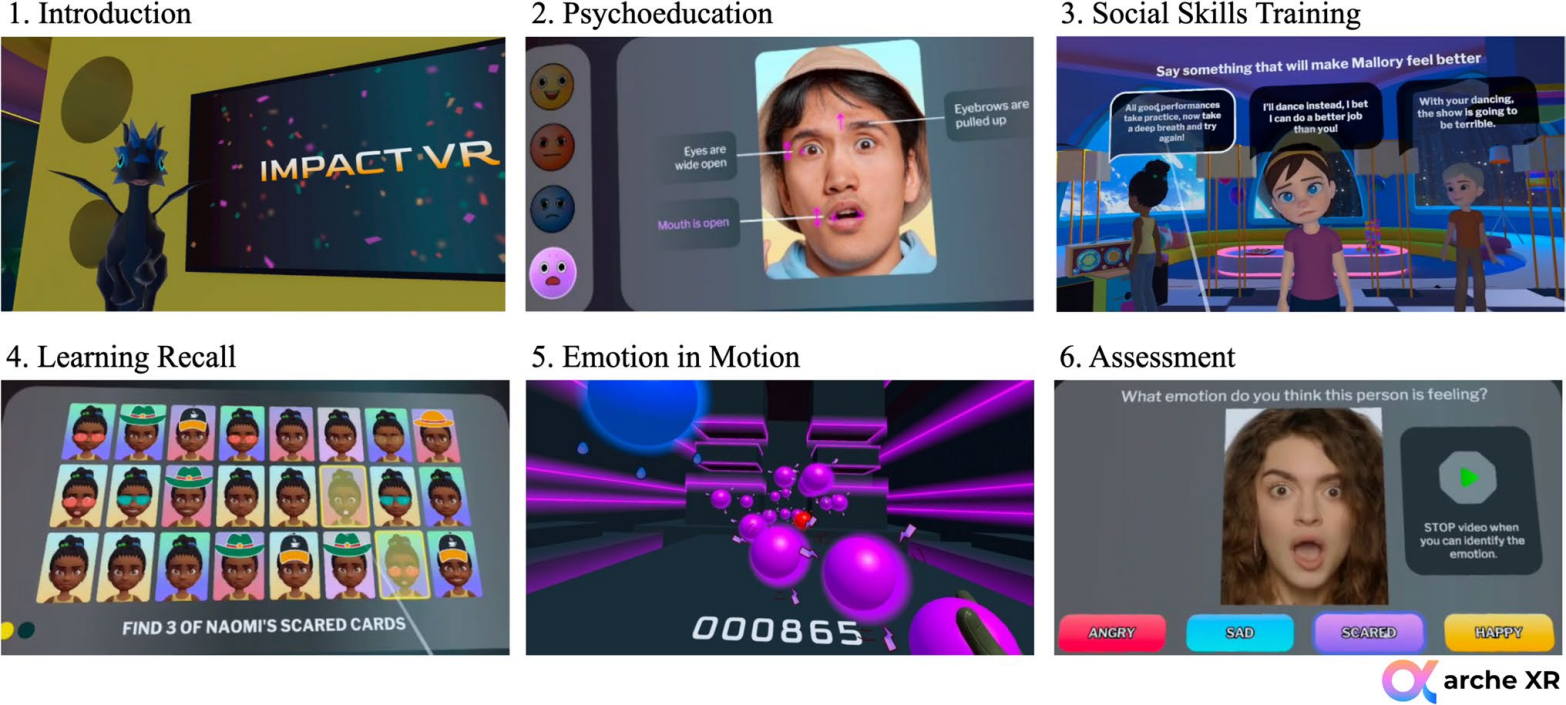
Session modules for impact VR

The Impact VR intervention is delivered across four ~ 25-min sessions, each designed to progressively build skills in emotion recognition, regulation, and prosocial behaviors. In Session 1 (Building the Foundations of Emotional Understanding), participants are introduced to core emotional expressions (happiness, sadness, anger, fear, and neutral) and learn to identify these emotions using facial features such as the eyes and mouth. Through mirroring tasks, static and dynamic identification, and scenario-based practice, youth receive immediate feedback to reinforce foundational skills. Session 2 (Enhancing Emotional Awareness and Adaptability) increases task complexity by presenting partially obscured facial cues (e.g., sunglasses or masks) and introduces basic emotion regulation strategies, helping participants recognize emotional triggers and practice appropriate responses in timed, gamified activities. Session 3 (Cultivating Emotional Connections in Social Settings) emphasizes applying emotional skills across social contexts as youth engage in a collaborative storyline involving peer interactions. Users practice interpreting emotional cues, resolving group conflicts, and responding empathetically to others’ feelings through interactive role-plays. In Session 4 (Applying Emotional Mastery in Complex Situations), participants apply all previously learned skills in more complex, emotionally charged scenarios involving multiple characters and overlapping emotional cues. They practice more advanced strategies to mitigate conflict and complete a final gamified task requiring real-time emotional interpretation and prosocial decision-making. Each Impact VR session follows a consistent structure to maximize engagement and skill acquisition. Sessions begin with an introduction from a virtual companion who outlines the session’s goals and reviews prior learning. This is followed by interactive psychoeducation activities focused on emotion recognition and regulation using both static and dynamic facial expressions, with immediate feedback provided. Youth then engage in social skills training through immersive, narrative-based scenarios that require identifying emotions, understanding their causes, and responding with prosocial behavior. A recall-based challenge reinforces key concepts before transitioning into the "Emotion in Motion" game, which is a physically active, music-synchronized task where participants match colored balls to emotional cues for points (see Fig. 2). For more information on Impact VR content and development, see Thomson et al., (2025a, b).

### Measures

#### Callous–Unemotional Traits (ICU)

The ICU (Kimonis et al., 2008) youth and caregiver versions were administered to assess CU traits in youth. The scale includes 24 items rated, which are rated on a Likert-scale (0 = Not at all true, 3 = Definitely true). The scale comprises both positively (e.g., “I do not show my emotions to others”) and negatively (e.g., “I am concerned about the feelings of others”) worded items; negatively worded items were reverse-scored, so higher scores signified higher levels of CU traits. The reliability and validity of the ICU has been validated in prior research (Pihet et al., 2015). The ICU yielded good internal consistency in the present study for both the youth and caregiver reports (α = 0.87; α = 0.84, respectively). ICU scores in the present study were similar (*M* = 25.60; SD = 9.30) to prior research with justice involved youth (*M* = 23.96; SD = 9.41; Kimonis et al., 2008) and adolescents with CD (*M* = 25.46; SD = 8.03; Elowsky et al., 2022), but higher than community-based school samples (e.g., *M* = 18.93, *SD* = 8.04; Kemp et al., 2023).

#### Conduct Problems

The Conduct Problems subscale from the BASC-3 Caregiver Rating Scales (Reynolds & Kamphaus, 2015) was used to assess caregiver-reported externalizing behavior. This subscale includes items rated on a 4-point Likert scale from 0 (Never) to 3 (Almost Always). The scale measures disruptive behaviors commonly associated with conduct problems, including defiance, rule-breaking, and aggression (e.g., “Breaks the rules,” “Lies or cheats”). T-scores were computed, with higher scores reflecting greater severity of conduct problem behaviors. In the current study, the Conduct Problems subscale demonstrated good internal consistency (α = .88). The caregiver reported Conduct Problems subscale yielded T-scores ranging from 37 to 95. The mean score of 57 falls just below the at-risk cutoff (T ≥ 60), consistent with elevated conduct problems (Reynolds & Kamphaus, 2015).

#### Reactive–Proactive Aggression Questionnaire (RPQ)

The RPQ (Raine et al., 2006) was administered to measure youth-reported aggression subtypes. The RPQ consists of 23 items rated on a 3-point scale from 0 (Never) to 2 (Often). It captures two distinct forms of aggression: reactive aggression (11 items; e.g., “Reacted angrily when provoked by others”) and proactive aggression (12 items; e.g., “Used force to obtain money or things from others”). Higher scores indicate a greater frequency of aggressive behavior. In the current study, both subscales demonstrated good internal consistency (reactive: α = .86; proactive: α = .84). Levels of reactive aggression in the present sample (*M* = 10.44, *SD* = 4.61) were consistent with those reported in a clinical sample of youth with CD (*M* = 9.12, *SD* = 5.52; Elowsky et al., 2022). Similarly, proactive aggression scores in the current study (*M* = 2.38, *SD* = 3.07) closely resembled those reported by Elowsky et al. (2022; *M* = 2.96, *SD* = 2.97).

### Power Analysis

A power analysis for the planned sample size of 110 participants assumed a 15% attrition rate per group and a correlation of 0.50 among repeated measures. Under these assumptions, the present study had 80% power to detect a medium effect size of *f* = 0.25 for the main effect of group at an alpha level of 0.05, assuming no interaction. In the presence of a statistically significant interaction, the study had 80% power to detect a Cohen’s *d* of 0.48 at an adjusted alpha of 0.025 to account for comparisons across the two follow-up time points. Both effect sizes are considered medium according to Cohen’s guidelines.

### Data Analytic Plan

Data was summarized using means and standard deviations for continuous variables and counts and proportions for categorical variables. We used linear mixed-effects models to test for differences in each outcome between Impact VR and the control condition. Models included fixed effects of group (Impact VR vs. Control), time (post-intervention vs. 3 months post-intervention), the group by time interaction, and the corresponding baseline score of the outcome. Additionally, the models included a random intercept to account for the within-subjects nature of the data. Our testing strategy was developed to maximize power and maintain familywise type I error rate. First, we tested the group by time interactions. If this was non-significant (*p* ≥ 0.05), we tested the marginal means of group using an alpha of 0.05 since there is insufficient evidence to conclude the effect of group is dependent on the time of measurement. In the event of a statistically significant interaction (*p* < 0.05), we tested the simple effect of groups within each time point using a Bonferroni adjusted alpha of 0.05/2 = 0.025 to account for 2 follow-up time points. For interpretability of effect sizes, we computed the standardized difference Cohen’s *d* using the baseline pooled variance for reach outcome and interpreted according to Cohen’s guidelines of 0.2, 0.5, and 0.8 for small, medium, and large effects, respectively (Cohen, 2013). Separate models were fit for each outcome using the lme4 package in the R statistical software (R Core Team, 2025). Table 2 presents the ANOVA results of the mixed models, and Table 3 presents the estimated means from mixed models.

**Table 2:** ANOVA tables from the results of the mixed models

Self-report CU					
	Mean Square	Num DF	Den DF	F	p
Group	328.91	1	98.81	7.15	0.009
Time	249.71	1	96.12	5.43	0.022
Group X Time	402.76	1	96.15	8.75	0.004
Baseline SR CU	2030.83	1	100.89	44.16	< 0.001
Caregiver-report CU					
	Mean Square	Num DF	Den DF	F	p
Group	545.37	1	101.31	18.28	< 0.001
Time	40.75	1	98.31	1.36	0.245
Group X Time	18.96	1	98.31	0.63	0.427
Baseline CG CU	1571.04	1	100.83	52.65	< 0.001
Conduct Problems					
	Mean Square	Num DF	Den DF	F	p
Group	299.92	1	93.39	15.74	< 0.001
Time	11.04	1	89.72	0.58	0.449
Group X Time	8.98	1	89.71	0.47	0.494
Baseline CP	387.38	1	93.31	20.33	< 0.001
Reactive Aggression					
	Mean Square	Num DF	Den DF	F	p
Group	169.50	1	95.10	17.21	< 0.001
Time	24.16	1	94.62	2.45	0.121
Group X Time	4.55	1	94.62	0.46	0.498
Baseline RA	184.81	1	95.97	18.76	< 0.001
Proactive Aggression					
	Mean Square	Num DF	Den DF	F	p
Group	5.55	1	91.27	2.95	0.089
Time	5.09	1	90.67	2.71	0.103
Group X Time	2.60	1	90.67	1.39	0.242
Baseline PA	73.75	1	93.01	39.27	< 0.001

**Table 3:** Estimated means from mixed models

Self-Report CU Post-Intervention				
Group	Mean	s.e	Lower	Upper
Control	24.8	1.17	22.5	27.1
Impact VR	24.2	1.13	21.9	26.4
	Mean	s.e	t	*p*
Difference	0.655	1.63	0.403	0.687
Self-Report CU 3 Months Post-Intervention				
Group	Mean	s.e	Lower	Upper
Control	25.5	1.21	23.1	27.9
Impact VR	19	1.19	16.6	21.3
	Mean	s.e	t	*p*
Difference	6.494	1.7	3.826	< 0.001
Caregiver CU Marginal Means				
Group	Mean	s.e	Lower	Upper
Control	27.9	1.24	25.5	30.4
Impact VR	20.4	1.24	18	22.9
	Mean	s.e	t	*p*
Difference	7.5	1.75	4.275	< 0.001
Conduct Problems Marginal Means				
Group	Mean	s.e	Lower	Upper
Control	55.8	1.37	53	58.5
Impact VR	48	1.39	45.2	50.7
	Mean	s.e	t	*p*
Difference	7.78	1.96	3.967	< 0.001
Reactive Aggression Marginal Means				
Group	Mean	s.e	Lower	Upper
Control	10.23	0.565	9.11	11.35
Impact VR	6.96	0.549	5.87	8.05
	Mean	s.e	t	*p*
Difference	3.27	0.79	4.148	< 0.001

## Results

### Callous-Unemotional Traits

**Fig. 3:**
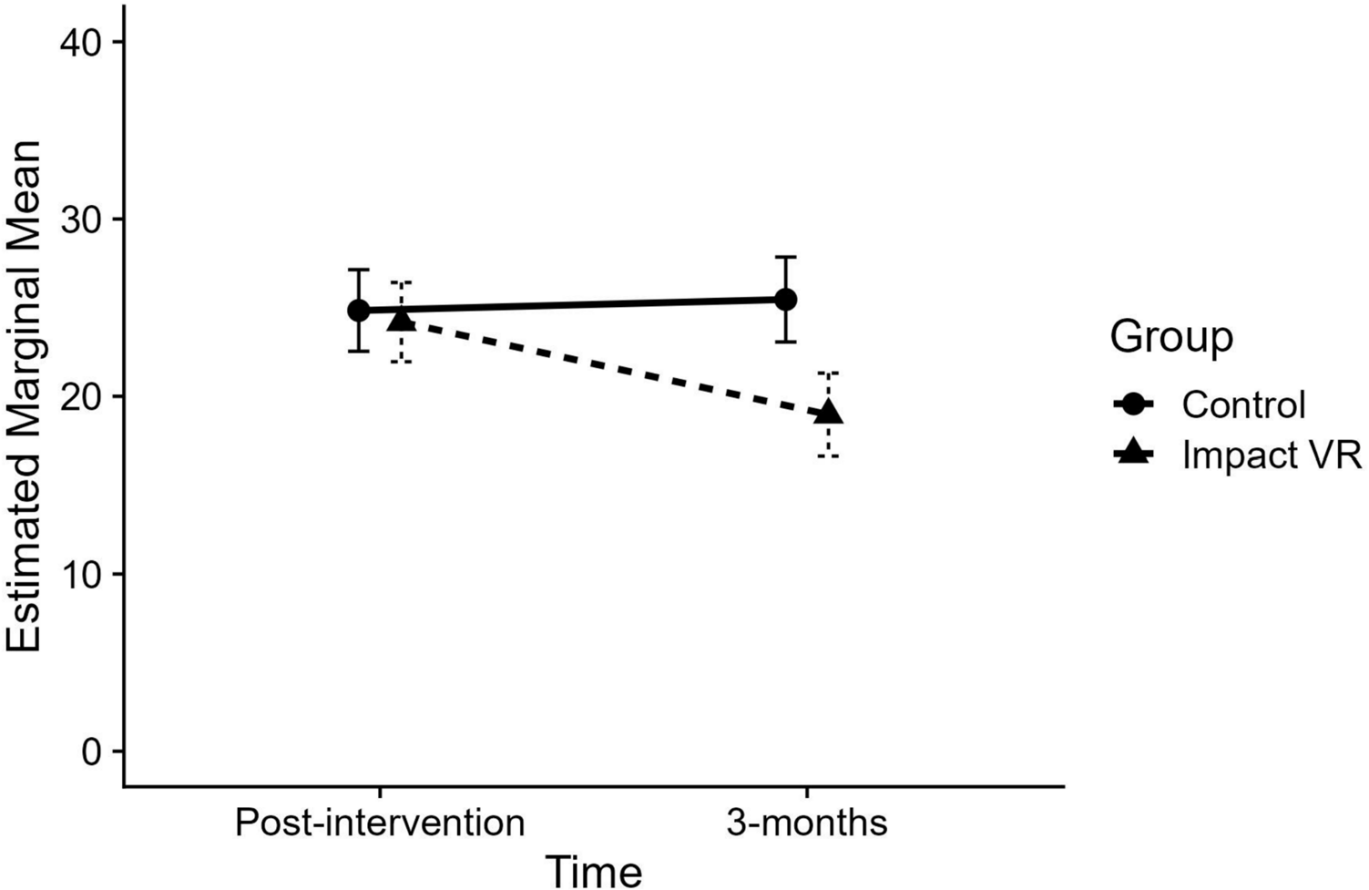
Estimated marginal means for self-reported CU traits at post-intervention and 3-month follow-up by group. Values reflect estimated marginal means adjusted for baseline scores. Baseline values are not shown. Horizontal offset applied for visual clarity only

When modeling self-reported CU traits, a significant group-by-time interaction emerged (*p* = .004), indicating that the change in CU traits over time differed by group. In addition, there were significant main effects of group (*p* = .008), and time (*p* = .022), suggesting that overall levels of CU traits differed by group and decreased over time. Given the significant group-by-time interaction, we examined the effect of group at each follow-up time point. At the 3-month post-intervention time point, a statistically significant difference existed between groups for self-reported CU traits (*p* < 0.001). The Impact VR group reported a mean score that was 6.5 units lower (*d* = 0.70) than the control group, indicating a medium to large improvement in CU traits at this time point. No statistically significant difference between groups was found immediately post-intervention (see Table 3 for results for all follow-up comparisons). Figure 3 displays the estimated marginal means and 95% confidence intervals for each group at post-intervention and 3-month follow-up, adjusted for baseline CU traits.

**Fig. 4:**
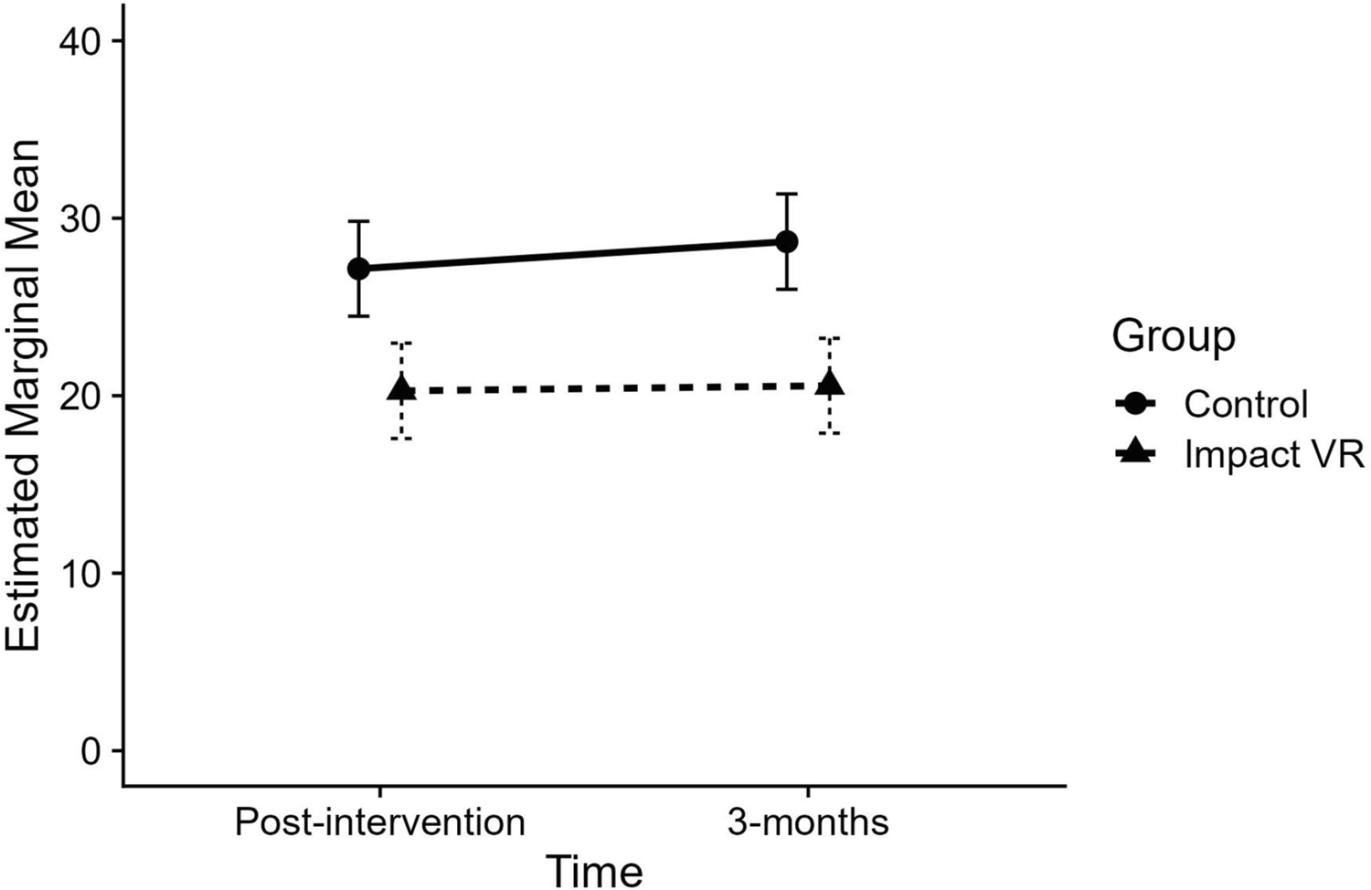
Estimated marginal means for caregiver-reported CU traits at post-intervention and 3-month follow-up by group. Values reflect estimated marginal means adjusted for baseline scores. Baseline values are not shown. Horizontal offset applied for visual clarity only

Caregivers of youth in the Impact VR group reported a mean 7.5 units lower CU traits score (*d* = 0.62) than that of the control group, indicating a medium-sized effect of the intervention (significant effects of group, *p* < 0.001). Neither the effect of time (*p* = 0.121) nor the group-by-time interaction (*p* = 0.498) was statistically significant, indicating that group differences were consistent over time and that CU traits were stable over time. Figure 4 displays the estimated marginal means and 95% confidence intervals for each group at post-intervention and 3-month follow-up, adjusted for baseline CU traits.

### Conduct Problems

The analysis of caregiver reports of youths’ conduct problems revealed statistically significant effects of group (*p* < 0.001) and baseline conduct problem scores (*p* < 0.001). There were no statistically significant effects of time (*p* = 0.245) or the group-by-time interaction (*p* = 0.427), indicating that the differences between groups and level of conduct problems did not vary over time. Examination of the marginal means indicated that the Impact VR group had conduct problem scores that were, on average, 7.78 units lower (*d* = 0.52) than those of the control group, indicating a medium-sized effect of the intervention.

### Reactive and Proactive Aggression

The model for reactive aggression revealed statistically significant effects of group (*p* < 0.001) and baseline reactive aggression scores (*p* < 0.001). However, neither the effect of time (*p* = 0.121) nor the group-by-time interaction (*p* = 0.498) was statistically significant. This indicates differences between groups and levels of aggression were stable over time. Marginal means showed that the Impact VR group had reactive aggression scores that were, on average, 3.27 units lower (*d* = 0.72) than those of the control group, indicating a medium to large effect of the Impact VR. The model for proactive aggression revealed only a statistically significant effect of baseline proactive aggression scores (*p* < 0.001). The effects of group (*p* = 0.089), time (*p* = 0.103), and their interaction (*p* = 0.242) were not statistically significant, providing insufficient evidence to support the effectiveness of Impact VR for reducing proactive aggression.

## Discussion

This study evaluated the efficacy of Impact VR, a brief, immersive VR intervention designed to improve socioemotional functioning in youth with CD. Findings demonstrated that youth with CD who received Impact VR had significant reductions in self-reported CU traits at the 3-month follow-up, with corroborating reductions in CU traits also reported by caregivers at both follow-up time points. In addition, there were decreases in overall conduct problems and reactive aggression at both time points for the Impact VR groups, though no effects were found for proactive aggression. These outcomes emerged following a time-limited intervention consisting of only four weekly sessions, suggesting that even brief, targeted exposure to emotionally salient, interactive learning environments can result in meaningful behavioral and emotional change. This study is among the first RCTs to demonstrate that CU traits, a core risk factor for persistent antisocial behavior, are malleable through intervention and that changes can occur by building protective factors, specifically, social skills training.

Traditional interventions for CD and externalizing behaviors have focused on behavior modification, parent management training, or cognitive-behavioral therapy. While these approaches yield modest success in improving conduct problems, their effects on CU traits have been limited. This is not surprising, as CU traits reflect deep-seated deficits in affective processing, empathic concern, and interpersonal attunement, which are personality traits that are not easily modified through behavior management-styled interventions (Thomson et al., 2017). The present findings suggest that targeting emotion recognition, a core socioemotional mechanism implicated in the developmental trajectory of CU traits, may offer a more direct and developmentally grounded route to intervention.

Youth high in CU traits are found to have deficits in recognizing fear, sadness, and distress in others (Fairchild et al., 2009; Marsh & Blair, 2008). These deficits are considered core to the social-emotional and moral impairments that define CU traits (Blair, 2013; Frick & White, 2008; R Blair, 2013; White et al., 2016). When youth cannot accurately perceive emotional cues, especially signs of vulnerability in others, they are less likely to develop affective empathy, to internalize social norms, or to experience guilt and remorse (Blair, 2013; Frick & White, 2008). Prior work suggests that youth with elevated CU traits may respond best to interventions focusing on emotional understanding and empathic processing, rather than general behavior management (Dadds et al., 2012; Thomson et al., 2017). In the present study, we show that a brief, emotion-focused intervention, delivered in an immersive, interactive format and rated as highly acceptable by youth with CD (Thomson et al., 2025a, b), can reduce CU traits over a short period. These findings directly challenge the assumption that CU traits are developmentally fixed or unresponsive to short-term, skills-based interventions.

Several features of Impact VR likely contributed to its efficacy. First, immersive VR provides a uniquely powerful way to simulate complex social situations in a controlled and repeatable manner. Unlike traditional computer-based or therapist-delivered training, VR engages users in dynamic environments that approximate real-life relational contexts. In doing so, it may tap into the same neural and affective systems that underlie live social interaction, allowing youth to practice interpreting and responding to emotional cues with immediacy and relevance. Second, the intervention was built from the ground up in collaboration with youth with CD, including those with high CU traits. By centering the voices and experiences of this population, Impact VR ensures that its tone, scenarios, and language are not only culturally relevant but also perceived as authentic. This “designed with” approach may be particularly important for youth with CU traits, who often disengage from interventions they perceive as moralizing, inauthentic, or irrelevant (Thomson et al., 2025a, b). The high levels of reported acceptability, even among high-risk youth with CD, challenge the assumption that youth with CD are unmotivated or unresponsive to interventions aimed at emotional development. Third, the VR format itself may have unique motivational and attentional benefits. Through reward-centered gameplay, VR may support learning of emotion recognition, social skills training, and emotion regulation tasks, which may also reduce perceived stigma and increasing willingness to engage. Unlike standard interventions where distraction, especially through phones or passive disengagement, is common, VR demands continuous attention and places the user at the center of the experience. In this way, VR does not just teach skills, it makes the emotional stakes of social interaction more immediate and more challenging to ignore.

CU traits are a reliable predictor of persistent antisocial behavior, including aggression, criminality, and violence (Pardini, 2006; Robertson et al., 2020; Vaughan et al., 2023). Thus, early interventions that can reduce these traits carry substantial public health value (Speck et al., 2024). Interestingly, the present study found reductions in CU traits observed in youth self-report at the 3-month follow-up but not immediately post-intervention. This delayed effect was not mirrored in caregiver reports, which showed improvement at both time points. One explanation is that youth high in CU traits may need more time and real-world feedback to recognize changes in their own emotional sensitivity or behavior. While caregivers can observe external shifts such as increased responsiveness or reduced conflict in daily interactions, youth may not immediately register these changes internally. This may reflect limited emotional awareness or a reduced tendency to reflect on interpersonal experiences, both of which are common among youth with CU traits. Impact VR may have initiated the process of emotional growth, but translating that growth into a change in self-perception likely requires more than brief exposure. For personality to shift, especially in domains related to empathy and emotional awareness, youth must encounter a variety of emotionally meaningful scenarios in which their new skills are practiced, reinforced, and reflected upon. This temporal lag in youth self-report is consistent with theories of social- learning, which emphasize that internalization of socioemotional skills depends on repeated application, social consequences, and experiential feedback across time and contexts (Bandura, 1973; Crick & Dodge, 1996). In this case, the intervention may have altered how youth interpret and respond to emotional situations, but they may only come to recognize these changes through continued engagement in real-life interactions over time. For youth with CU traits, that recognition is likely to emerge gradually as behavioral patterns shift and the social feedback they receive begins to differ in consistent and meaningful ways. In contrast, youth did report immediate reductions in reactive aggression, a behavioral outcome that was also supported by caregiver report of reduced conduct problems. These parallel findings suggest that although internal changes in callousness, empathy, and emotionality may take time to change, behavior can shift more quickly when youth are given the tools to navigate conflict and regulate their responses in emotionally charged situations.

The lack of significant findings for proactive aggression should be interpreted with caution due to floor effects. Baseline scores were low across both groups (means < 2.5), with standard deviations exceeding the means, suggesting limited variability in the sample. Although these scores are similar to other adolescent CD samples (e.g., Elowsky et al., 2022), there is little that can be concluded about the intervention’s impact on proactive aggression in this context.

### Limitations

While this study's results are promising, several limitations should be considered. First, although multiple informants were used, the primary outcome of CU traits relied on youth self- and caregiver reports. While these surveys are often the most direct way to assess CU traits, it is important to understand if reductions in CU traits reduced clinical levels of CU to sub-clinical levels. Thus, future studies should include a semi-structured assessment of CU traits (i.e., CAPE; Frick, 2013). Relatedly, although the present study participants had similar ICU scores to prior research involving adolescents with CD (Elowsky et al., 2022) and older samples of justice-involved youth (Kemp et al., 2023; Robertson et al., 2021), the scores are lower than some research involving forensic and stratified samples. This may limit the generalizability of the results. Second, while the present study suggests improvements in CU, CP, and aggression were sustained, it remains unknown for how long these effects impact youth. Therefore, longer-term follow-up is essential to determine whether reductions in CU traits, CP, and aggression remain, attenuate, or increase over time. It is also possible that other benefits may emerge later, particularly in domains such as peer relationships, academic functioning, or involvement in the justice system. Third, while the immersive nature of VR is a strength, it also introduces the possibility of novelty effects. That is, youth may initially engage with the program simply because it is new or technologically appealing. However, the fact that effects were delayed, not immediate, argues against a pure novelty explanation and suggests that learning and behavioral consolidation occurred over time. Fourth, findings from this study point to emotion recognition and social skills development as likely mechanisms driving the observed intervention effects. However, future research with larger samples is needed to formally test these mechanisms and to examine potential biopsychosocial moderators and mediators. Similarly, identifying for whom and how the intervention works will be essential for refining its implementation and targeting efforts toward the youth most likely to benefit. Lastly, although the control condition replicated a one-session emotion recognition training shown to improve outcomes among youth with CU traits and conduct problems (Dadds et al., 2012; Hunnikin et al., 2022; Muñoz Centifanti et al., 2021), it involved a lower treatment dosage than Impact VR’s four 25-min sessions. Given these encouraging findings, a larger-scale study should evaluate the effectiveness of Impact VR relative to more comprehensive evidence-based approaches, such as CBT or family-based interventions (e.g., functional family therapy). Importantly, due to its low cost and minimal resource demands, it may also be valuable to explore how Impact VR can enhance existing treatments to achieve greater gains in youth outcomes.

This study represents a Phase II clinical trial of Impact VR, following prior Phase I research demonstrating its acceptability, feasibility, and usability among key stakeholders (Thomson et al., 2025a, b). By evaluating preliminary efficacy in a randomized sample of 110 youth with CD against an active comparator, this Phase II trial provides promising early evidence for reductions in CU traits, conduct problems, and aggression. However, consistent with the clinical trial development process, a fully powered Phase III trial is needed to rigorously evaluate effectiveness relative to established evidence-based treatments (e.g., CBT, family therapy), examine long-term clinical and objective outcomes (e.g., criminal and school infractions), and assess generalizability across diverse clinical and forensic settings.

## Conclusion

This study provides novel evidence that CU traits can be reduced through a brief, developmentally grounded, emotionally focused VR intervention. Impact VR leverages immersive technology to deliver targeted emotion recognition and social skills training content in a format that is engaging, accessible, and scalable. This represents a critical step forward for youth with CD, particularly those with CU traits. Rather than focusing solely on external behaviors, we argue that interventions must engage the internal socioemotional processes that drive those behaviors. When these mechanisms are addressed directly, and in a way that youth are willing to engage with, meaningful change is possible. This study underscores the importance of innovation in developmental and clinical science.

## Data Availability

The study dataset is not publicly available due to privacy restrictions, but permission may be granted from the corresponding author upon reasonable request and a data use agreement (DUA).
